# Fundraising in Education: Road Map to Involving Medical Educators in Fundraising

**DOI:** 10.2196/32597

**Published:** 2022-04-05

**Authors:** Alireza Jalali, Jacline A Nyman, Elaine Hamelin-Mitchell

**Affiliations:** 1 Faculty of Medicine University of Ottawa Ottawa, ON Canada

**Keywords:** fundraising, philanthropy, crowdfunding, funding, charity, higher education, university, business model, revenue streams, medical education, educators, academia, academic environments

## Abstract

Traditional funding models must change as governments decrease funding and often freeze tuition at a domestic level. As a result, universities face an increasing need to diversify their business models, including revenue streams. Therefore, interest in raising significant funds from other sources is stronger than ever, leading to the need for a fundraising approach that is more sophisticated. Medical educators and health professionals are some of the most trusted members of society, and with this paper, the authors aim to raise awareness of the critical role they play in helping universities with their global impact and fundraising efforts.

## Introduction

This is a time of economic, societal, and political challenges in higher education. Therefore, universities today focus more than ever on fundraising. To diversify their traditional revenue streams, universities are increasing their international student recruitment efforts and are expanding their research boundaries as global actors and agents of change. Therefore, interest in fundraising from private sources is stronger than ever, leading to the need for a fundraising approach that is more sophisticated. As such, medical educators must become agents of change and reflect on strategies to ensure the successful implementation of fundraising programs in academic environments toward achieving maximum impact. *Fundraising* is defined as seeking financial support for a nonprofit organization or cause. However, in the Greek language, the word *philanthropy* means “love [Philos] of humanity [Anthropos].” Because philanthropy fuels the act of fundraising, fundraising is not only about raising money through tactics and transactions but also about changing lives, making an impact, and cultivating long-lasting and meaningful relationships with donors and the community.

## Defining a Fundraising Road Map

**Figure 1:**
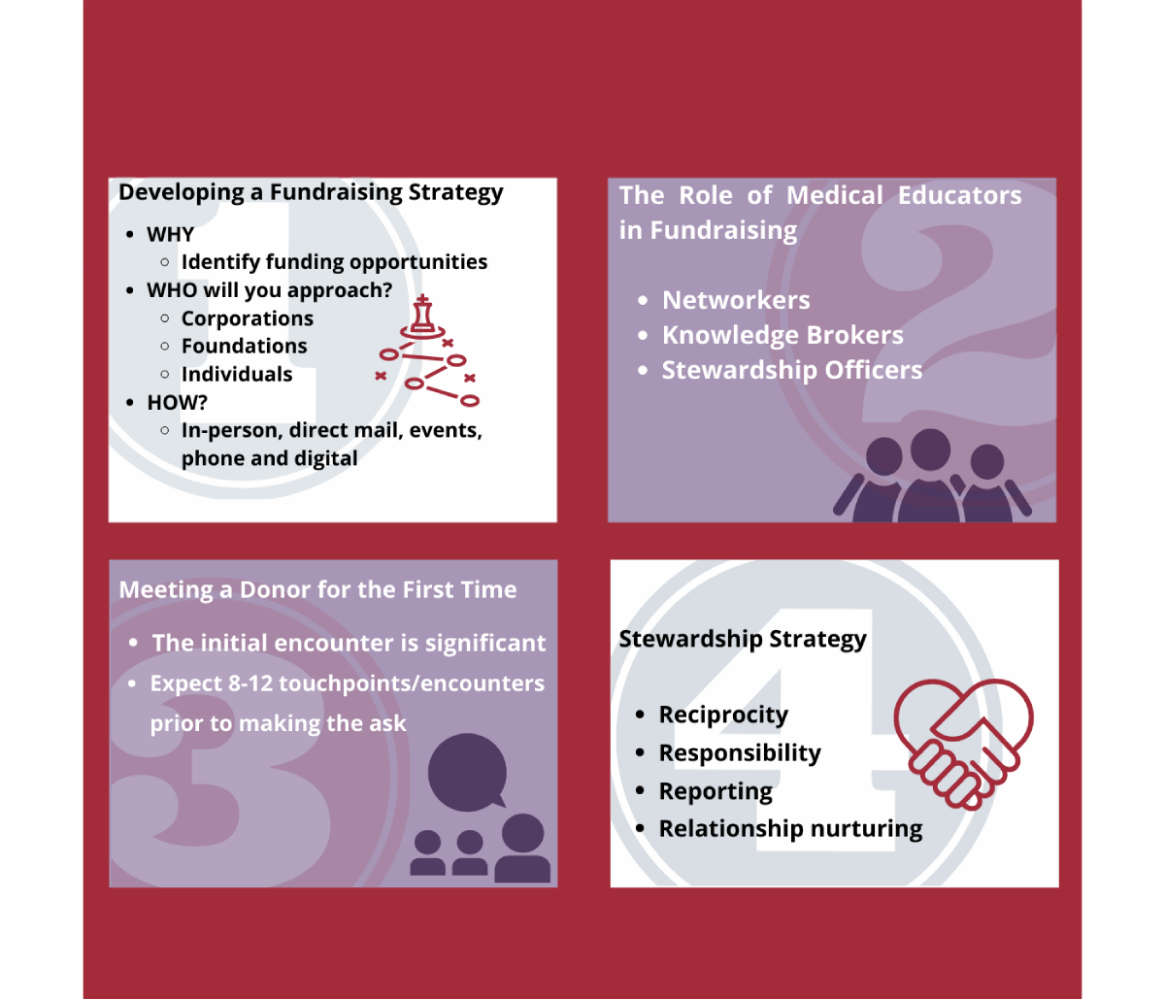
The fundraising road map.

The first step on this journey is to develop a *fundraising road map*: a plan of action that guides an institute’s fundraising activities (Figure 1). A road map sets out the fundraising objectives and the ways the institute will meet them. Fundraising objectives should align with the institute’s strategic plan and areas with the most significant impact. A fundraising plan should also consider donors’ needs by connecting them to benefits that affect them, such as life-changing clinical research.

To develop a successful road map, institutions need more than a vision and a strategic plan. Educators need to thoroughly understand their budgets, knowing why they need the funds and how much they need in advance. As such, some basic knowledge of fundraising is essential to any medical educator venturing into this field. First, the road map needs to be adapted to the amount of financial support required to fund a given project successfully. A sophisticated fundraising program will include an annual fund or a fundraising event aiming to acquire donors through a tactical approach typically aimed at raising funds up to US $25,000. Relationship-oriented major gift fundraising programs require significant development of authentic longer-term relationships with donors. These programs aim to raise gifts of US $25,000 or more, including top-level “principal” gifts of US $1 million or more, and even transformational giving that exceeds US $5 million. Donors may also consider leaving significant planned gifts, whether during their lifetimes or upon death, as part of an overall financial and estate plan. Gifts in kind, where goods and services are given as donations, are another option. Each of these types of fundraising programs requires a well-established fundraising strategy.

For example, a researcher approaches you with a crucial need for incubators for the new Advanced Medical Research Centre. After careful budget analysis, it appears there are insufficient funds to acquire this equipment. Estimating that a state-of-the-art incubator costs roughly US $30,000, you can begin to build a “major gift” fundraising campaign plan. However, if researchers require multiple incubators, you may need to target “principal” level donations depending on the amount. The key here is to clarify and confirm the exact funding requirement and align it to the giving capacity of your potential donors.

## Developing a Fundraising Strategy

Your strategy defines who you will approach and in what way. This can be achieved by conducting a funding gap analysis to determine the amount needed to fund your project’s ongoing operations or future development and to match interested donors to suitable initiatives. Most donations come from three types of donors: corporations (eg, corporate matching gift programs and volunteer grants), foundations (eg, grants from nongovernmental organizations), and individuals. Each of these donor types can be approached through any of the five main types of fundraising: in-person, direct mail, events, phone (SMS text messaging), and digital [1]. Recent studies have shown social media also plays an important role in fundraising, mostly in crowdfunding campaigns [2,3]. However, for major gift fundraising, personal, one-on-one solicitation remains the most successful means of gift solicitation.

The development of a well-researched fundraising strategy is essential to the success of one’s efforts, and fund raisers must decide on donors and approaches carefully based on their needs and environment.

## The Role of Medical Educators in Fundraising

Medical educators can become an essential part of the fundraising team because they provide a unique perspective from within the faculty. As expert witnesses to a fundraising strategy, they can elaborate on the faculty’s vision and goals, provide details of programs, explain the impact of medical research, and often have the most significant working relationships with potential donors (this is key to prospect identification). Participating in fundraising expands upon their existing role of expert witnesses for their work or research funding [4].

Medical educators can play three roles in fundraising [5], which are as follows: (1) networker—by networking with patients, clinicians, researchers, and other educators, they can serve as a connection between the fundraising team and the groups mentioned above, as well as open doors within the medical community; (2) knowledge broker—as knowledge brokers, medical educators can serve as the fundraising team’s academic lead and can provide access to knowledge about the faculty’s strategic priorities, research innovations, and medical advances. In this way, they can assist the fundraising team in establishing links between donor interests and the strategic preferences of the institute; and (3) stewardship officer—in the capacity of stewardship officers, medical educators can meet with donors and alumni to update them on the latest institutional achievements and answer their questions.

## Meeting a Donor for the First Time

The first encounter with a donor is significant. A few essential tips on a successful first meeting include engaging the prospect and listening more than talking about them. The process should focus on donors instead of the individuals doing the fundraising. Make a note of how many times you use the word “I” versus “you.” The conversation should always be “you” dominant. During your encounters, always be enthusiastic, authentic, and passionate. Discuss your strengths, and bring along colleagues who might be able to help in areas of uncertainty. Always take notes (with permission) and end meetings by establishing the next steps. Typically, it takes 12-18 months to develop meaningful relationships that result in asking prospective donors to give, and within that period, 8-10 significant “moves” or actions, including in-person meetings, can occur. When ready to ask for a donation, ask for a specific amount and keep your sights high. The amount asked should be based on both the donor’s wealth and the value of the project. Praise other peoples’ commitments to your program without breaching any confidentiality.

## Stewardship Strategy

Lastly, develop a customized stewardship strategy for the donor, which means having a systematic approach to cultivate and improve your relationships with donors. The four stewardship strategies are reciprocity (must demonstrate gratitude), responsibility (must act in a socially responsible manner), reporting (need to keep its public informed), and relationship nurturing (should make sure donors receive thank you letters and annual reports and are invited to special events). As the relationship strengthens, fundraisers may also send handwritten cards for special occasions [6].

## Ethical Practice in Fundraising

A critical point for any medical educator venturing into fundraising is always to follow strict ethical rules regarding interaction with patients and learners. Unwarranted pressure on patients or learners to contribute should be avoided, and patient or learner confidentiality and trust should always be a priority. The main recommendation is for institutions to have a fundraising committee made of educators, physicians, and fundraisers to mitigate these risks and for fundraisers to follow ethical guidelines established by their organization [7].

In conclusion, in this modern age, having medical educators collaborate closely with fundraising teams is essential. Educators serve as the link between the donor, the fundraiser, and the life-changing medical advances toward which we work. As academic leaders and subject matter experts, medical educators bring imperative knowledge and connections vital to any fundraising campaign’s success.
